# miR-488-3p Protects Cardiomyocytes against Doxorubicin-Induced Cardiotoxicity by Inhibiting CyclinG1

**DOI:** 10.1155/2022/5184135

**Published:** 2022-02-10

**Authors:** Mingjing Yan, Yuan Cao, Que Wang, Kun Xu, Lin Dou, Xiuqing Huang, Beidong Chen, Weiqing Tang, Ming Lan, Bing Liu, Kaiyi Zhu, Yao Yang, Shenghui Sun, Xiyue Zhang, Yong Man, Mingyan Hei, Tao Shen, Jian Li

**Affiliations:** ^1^Peking University Fifth School of Clinical Medicine, Beijing 100730, China; ^2^The Key Laboratory of Geriatrics, Beijing Institute of Geriatrics, Institute of Geriatric Medicine, Chinese Academy of Medical Sciences, Beijing Hospital/National Center of Gerontology of National Health Commission, Beijing 100730, China; ^3^Department of Neonatology, Neonatal Center, Beijing Children's Hospital, Capital Medical University, Beijing 100045, China

## Abstract

**Objective:**

To investigate the protective effects and regulatory mechanism of miR-488-3p on doxorubicin-induced cardiotoxicity.

**Methods:**

The C57BL/6 mice and primary cardiomyocytes were used to construct doxorubicin-induced cardiomyocyte injury models in vivo and in vitro. The levels of miR-488-3p and its downstream target genes were analyzed by quantitative real-time PCR. Mouse cardiac function, cell survival, cellular injury-related proteins, and the apoptosis level of cardiomyocytes were analyzed by echocardiography, MTT analysis, Western blotting, and DNA laddering separately.

**Results:**

Cardiomyocyte injury caused by a variety of stimuli can lead to the reduction of miR-488-3p level, especially when stimulated with doxorubicin. Doxorubicin led to significant decrease in cardiac function, cell autophagic flux blockage, and apoptosis in vivo and in vitro. The expression of miR-488-3p's target gene, CyclinG1, increased remarkably in the doxorubicin-treated neonatal mouse cardiomyocytes. Overexpression of miR-488-3p inhibited CyclinG1 expression, increased cardiomyocyte viability, and attenuated doxorubicin-induced cardiomyocyte autophagic flux blockage and apoptosis.

**Conclusions:**

miR-488-3p is one of the important protective miRNAs in doxorubicin-induced cardiotoxicity by inhibiting the expression of CyclinG1, which provides insight into the possible clinical application of miR-488-3p/CyclinG1 as therapeutic targets in doxorubicin-induced cardiovascular diseases.

## 1. Introduction

Although cancer incidence and mortality have decreased in recent years, they still pose a serious threat to various physiological and behavioral processes. Statistics show that the number of new cases of cancer remains a challenge for the medical community each year [1]. Cancer is one of the leading causes of global death [2]. The high incidence of cancer and high mortality also contributed to the growth of the anticancer drug market.

Doxorubicin is an efficient antineoplastic drug and can play a significant role in the treatment of solid tumors, leukemia, and lymphoma. However, doxorubicin is limitedly used in clinic for its cardiotoxicity [3–5]. The molecular mechanisms of doxorubicin-induced cardiotoxicity are widely accepted as follows: (I) oxidative stress [6, 7]; (II) DNA damage: doxorubicin inhibits the activity of topoisomerase in cardiomyocytes [8]; (III) mitochondrial damage [9]; (IV) autophagic flux blockage [10, 11]; (V) apoptosis and necroptosis [12, 13]; and (VII) disturbance of calcium homeostasis [14]. However, the underlying mechanism is not completely clear in the pathogenesis of doxorubicin-induced cardiotoxicity. Therefore, it is of great clinical significance to study the mechanism and inhibit or alleviate doxorubicin-induced cardiotoxicity.

MicroRNAs (miRNAs), evolutionarily conserved noncoding RNA molecules with 18-25 nucleotides, are negatively posttranscriptional gene regulators [15] and are involved in the development of cardiovascular disease [16]. Studies demonstrate that miR-488-3p is downregulated in papillary thyroid cancer [17] and colorectal cancer [18] and plays a tumor-suppressive role in nearly all cancers [19]. miR-488-3p also inhibits cell proliferation and migration by targeting DCX in Hirschsprung's disease [20]. Recent studies have described that the long noncoding RNA (lncRNA) Cerox1 can regulate the catalytic activity of mitochondrial complex I by binding to miR-488-3p, which in turn regulates the level of mitochondrial oxidative phosphorylation and reduces the production of reactive oxygen species [21]. However, the role of miR-488-3p in doxorubicin-induced cardiotoxicity is still not clear.

In this study, we investigated the function and regulation mechanism of miR-488-3p in doxorubicin-induced myocardial injury, to provide a theoretical basis for the prevention and treatment of cardiotoxicity reduced by doxorubicin.

## 2. Materials and Methods

### 2.1. Reagents

Doxorubicin was purchased from Cell Signaling Technology (Danvers, MA, USA). LC3B antibody was purchased from Sigma-Aldrich (St. Louis, MO, USA); caspase-3 antibodies were purchased from Santa Cruz (Dallas, TX, USA) and Cell Signaling Technology (Danvers, MA, USA); p62 antibody and horseradish peroxidase- (HRP-) conjugated secondary antibody were purchased from Cell Signaling Technology (Danvers, MA, USA); CyclinG1 antibody was purchased from Proteintech Group (Chicago, IL, USA). Unless otherwise specified, all other chemicals were purchased from Sigma (St. Louis, MO, USA) or Solarbio (Beijing, China).

### 2.2. Animals and Treatments

Ten-week-old male C57BL/6 mice were purchased from SPF Biotechnology Co., Ltd. (Beijing, China) and fed in a platform of specific pathogen free at constant temperature (22°C) with 12 hours of light-dark circulation and unlimited diet and water. Mice were randomly divided into control group and doxorubicin treatment group. Doxorubicin (7.5 mg/kg) or saline was delivered to mice every other day for a total of three injections by intraperitoneal injection. Cardiac function was measured after 6 days of doxorubicin injection, and then, the hearts were harvested for further analysis. All animal experiments were approved by the Peking University Animal Use and Care Committee and conducted according to the Guide for Care and Use of Laboratory Animals (NIH Publication # 85-23, revised 1996).

### 2.3. Cell Culture and Treatments

Neonatal mouse ventricular myocytes (NMVMs) were isolated from 1-3-day-old C57BL/6J mice by digestion with the combination of trypsin and collagenase type II as reported previously [16]. After the hearts were digested completely, cardiac myocytes were filtered with mesh and passed onto dishes. Two hours later, floating cells were passed onto 6-well culture plates at a density of 6.3 × 10^5^ cells/well with M-199/DMEM media mix supplemented with 5% fetal bovine serum (FBS) and 10% horse serum in the presence of 0.1 mM 5-bromo-2-deoxyuridine and incubated at 37°C in humidified air with 5% CO_2_. The NMVMs were cultured for 36 hours, and serum was withdrawn from the medium for 24 hours. After serum starvation, the cells were treated with doxorubicin for 24 hours.

The sequences of negative control, miR-488-3p mimics, microRNA inhibitor negative control, and miR-488-3p inhibitor were as follows (5′–3′): negative control sense, UUCUCCGAACGUGUCACGUTT; negative control antisense, ACGUGACACGUUCGGAGAATT; miR-488-3p mimics, UUGAAAGGCUGUUUCUUGGUC and CCAAGAAACAGCCUUUCAAUU; microRNA inhibitor negative control, CAGUACUUUUGUGUAGUACAA; and miR-488-3p inhibitor, GACCAAGAAACAGCCUUUCAA. miRNA oligos were purchased from GenePharma Co. (Suzhou, China). miRNA mimics and inhibitors were transfected using the Lipofectamine RNAiMAX Transfection Reagent (Invitrogen, CA, USA) according to the manufacturer's protocol. After 8 hours of transfection, the medium was replaced with fresh medium. Then, the cells were cultured for 36 hours before doxorubicin treatment.

### 2.4. RNA Extraction and Real-Time PCR

Total RNAs were extracted from left ventricles or cardiomyocytes using TRIzol (Invitrogen, Carlsbad, CA, USA). The reverse transcription of RNA into cDNA was performed using the first-strand cDNA synthesis kit (New England Biolabs). The reaction systems containing SYBR green (TaKaRa, Japan) were prepared according to instructions. Real-time PCR was performed using the QuantStudio3 Real-Time PCR system (Thermo Fisher Scientific, US) as described previously [22]. The miR-488-3p reverse transcription primer is GTCGTATCCAGTGCAGGGTCCGAGGTATTCGCACTGGATACGACGACCAAG, the miR-488-3p PCR 5′ primer is CATCATCGTTGAAAGGCTGTT, the U6 reverse transcription primer is GTCGTATCCAGTGCAGGGTCCGAGGTATTCGCACTGGATACGACAAATATG, the U6 PCR 5′ primer is GCGCTCGTGAAGCGTTC, the Universe 3′ primer is GTGCAGGGTCCGAGGT, the mouse CyclinG1 5′ primer is CGAGCTCGCAGAGTCTCCATGCTGGCTT, and the mouse CyclinG1 3′ primer is CCGCTCGAGCGGTTGATAACTGCACTACTAAGAG. The data was the average of 3-6 independent experiments.

### 2.5. Luciferase Assay

The 3′-UTR fragment of CyclinG1 containing the miR-488-3p binding site was amplified by PCR. The fragment was inserted into the pmir-GLO luciferase reporter vector (Promega, USA) as described previously [17]. The PCR product fragments and pmirGLO plasmids were digested with SacI and XhoI. The digested fragments and vectors were ligated to construct recombinant plasmids. Then, recombinant plasmids were transformed into *E. coli* DH5*α* competent cells, coated on a LB plate containing 1% Ampicillin, and cultured overnight at 37°C. The recombinant monoclonal colonies were selected and amplified in LB liquid medium with 1% Ampicillin. The positive cloned plasmids were selected and sent to Sangon Biotech (Shanghai, China) for further sequencing verification. For the luciferase assay, miR-488-3p mimics were cotransfected with the luciferase reporters containing CyclinG1 3′-UTR or pmir-GLO luciferase reporter vector into HEK-293A cells using a transfection reagent (Vigofect, Vigorous Biotechnology, China). Forty-eight hours after transfection, luciferase activity was detected using the Dual-Luciferase Reporter Assay System (Promega).

### 2.6. Echocardiography

Mice were mildly anesthetized with 1-1.5% isoflurane until their heart rate stabilized to 400 to 500 beats per minute while maintaining their normal body temperature. Echocardiography was performed with Vevo 770 and Vevo 2100 (VisualSonics) instruments. The left ventricular anterior wall (LVAW), left ventricular posterior wall (LVPW), ejection fraction (EF%), and fraction shortening (FS%) were calculated using Vevo Analysis software (version 2.2.3) as described previously [22].

### 2.7. Histology Staining

Histology assays were performed on adult mouse heart sections. The hearts were processed as paraffin-embedded sections and were subsequently analyzed by H&E staining according to the manufacturer's protocol (Sigma-Aldrich) as described previously [16].

### 2.8. *In Situ* Apoptosis Detection

The CardioTACS™ *In Situ* Apoptosis Detection Kit (4827-30-K) was obtained from R&D Systems, Inc. As previously described, the cultured cardiomyocytes or cryosection frozen tissue sections were fixed with 4% paraformaldehyde for 10 minutes and then washed with PBS. The cell membranes were permeabilized with the Cytonin™ reagent for 30 minutes at room temperature. Endogenous peroxidase activity was quenched with 5% hydrogen peroxide (H_2_O_2_) for 5 min at room temperature. Next, cover the samples with the Terminal deoxynucleotidyl Transferase (TdT) Labeling Reaction Mix, and incubate them at 37°C for 1 hour in a humidity chamber. Wash samples 2 times in distilled water, and incubate them with Streptavidin-HRP Working Solution for 10 minutes at room temperature. The samples were counterstained with Nuclear Fast Red for 5 minutes. The stained samples were observed with a light microscope.

### 2.9. MTT Assay

The viability of neonatal mouse cardiomyocytes treated with different concentrations of doxorubicin was detected with the MTT (3-(4,5-dimethylthiazol-2-yl)-2,5-bromide) assay kit (Roche Applied Science, Indianapolis, IN) according to the manufacturer's instructions as described previously [23].

### 2.10. DNA Laddering

The cultured cells (5 × 10^6^ cells per each plate) were lysed in lysis buffer lysate containing 10% SDS and 100 g/mL protease K and incubated overnight at 37°C. DNA was extracted with phenol/CHCl_3_/isoamyl alcohol and CHCl_3_/isoamyl alcohol, respectively. The extracted DNA was stored at -20°C for one week after adding sodium acetate and ethanol. DNA fragments were detected by agarose gel electrophoresis and visualized by ethidium bromide staining.

### 2.11. Monodansylcadaverine (MDC) Staining

Autophagic vesicles in response to different treatments were analyzed by using the monodansylcadaverine (MDC) kit (Solarbio, Beijing, China). After treatments, the cardiomyocytes were stained with MDC following the manufacturer's protocol. Briefly, 300 *μ*L of the staining solution was added to the cells and incubated for 45 minutes at room temperature. Then, the cells were washed twice with washing buffer and examined by fluorescence microscopy with a 355 nm excitation wavelength and a 512 nm emission filter. The intensity of immunofluorescence staining was quantified using ImageJ software (NIH, Maryland, USA).

### 2.12. Western Blotting Analysis

Cardiomyocytes or left ventricles were lysed with lysis buffer containing a complete protease inhibitor and phosphatase inhibitor. Total proteins were separated by sodium dodecyl sulfate polyacrylamide gel electrophoresis (SDS-PAGE) and electrotransferred to polyvinylidene difluoride (PVDF) membranes. The membranes were then blocked with 5% skim milk at room temperature for 2 hours and incubated with specific antibodies overnight at 4°C. After being washed with Tris-buffered saline containing 0.1% Tween 20 for 1 hour, the membranes were incubated with a horseradish peroxidase-conjugated secondary antibody for 1 hour at room temperature. Bands were detected by using chemiluminescence detection reagents and analyzed by using ImageJ software.

### 2.13. Statistical Analysis

All statistical calculations were performed using GraphPad Prism 8 software. All data are presented as means ± SEM. The difference between the two groups was analyzed by the unpaired *t*-test, and multiple comparisons were analyzed by one-way ANOVA with the Bonferroni correction. *P* values < 0.05 were considered significant. ^∗^*P* < 0.05, ^∗∗^*P* < 0.01, and ^∗∗∗^*P* < 0.001 in Student's *t*-test.

## 3. Results

### 3.1. miR-488-3p Is Downregulated in Cardiomyocyte Injury

**Figure 1:**
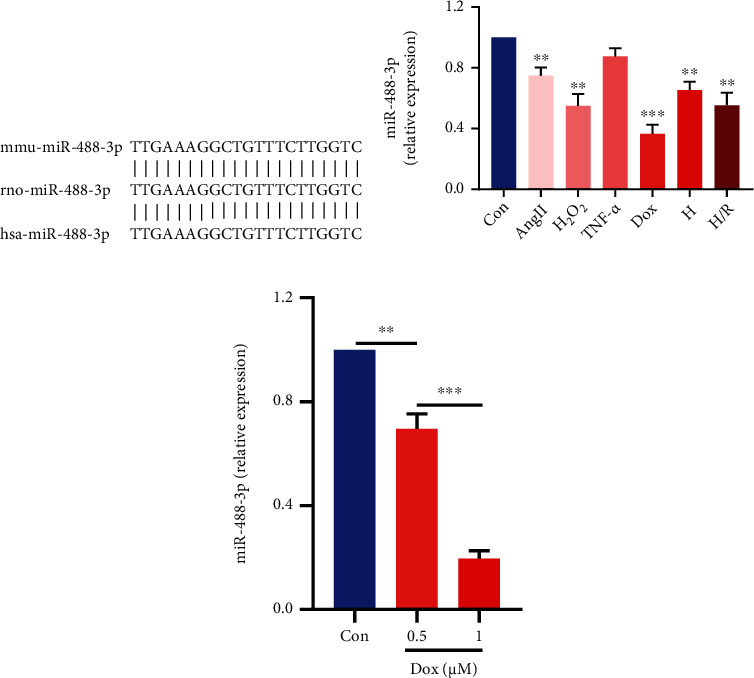
Downregulation of miR-488-3p is correlated with myocardial injury. (a) miR-488-3p sequences in the mouse, rat, and human for the evolutionary conservation analysis. (b) The changes in miR-488-3p expression in different stimulus-induced myocardial injury models (*n* = 3). (c) Identification of miR-488-3p expression in cardiomyocytes treated with 0.5 *μ*M and 1 *μ*M doxorubicin (*n* = 3). ^∗∗^*P* < 0.01, ^∗∗∗^*P* < 0.001.

miR-488-3p is highly conservative in mouse, rat, and human and is abundantly expressed in the heart (see Figure 1(a)). To investigate the function of miR-488-3p on cardiomyocytes, a number of the primary mouse ventricular cardiomyocyte injury models were established, such as angiotensin II-, H_2_O_2_-, TNF-*α*-, doxorubicin-, hypoxia-, and hypoxia-reoxygenation-induced cardiomyocyte injury models. The results showed that almost all the stimulations, except TNF-*α*, could decrease miR-488-3p expression in cardiomyocytes (see Figure 1(b)). And doxorubicin was the most significant stimulator of decreased miR-488-3p among all the cardiomyocyte injury models in our study (see Figure 1(b)).

The level of miR-488-3p was analyzed in control and doxorubicin-treated cardiomyocytes. Remarkably, with the increase in doxorubicin, the expression of miR-488-3p was decreased simultaneously in cardiomyocytes in a concentration-dependent manner (see Figure 1(c)). Therefore, we proved that the downregulation of miR-488-3p is correlated with the cardiomyocyte injury and speculated that doxorubicin might be one of the important pathological stimulators regulating miR-488-3p expression.

### 3.2. miR-488-3p Expression Is Downregulated in Doxorubicin-Induced Cardiomyopathy In Vivo

**Figure 2:**
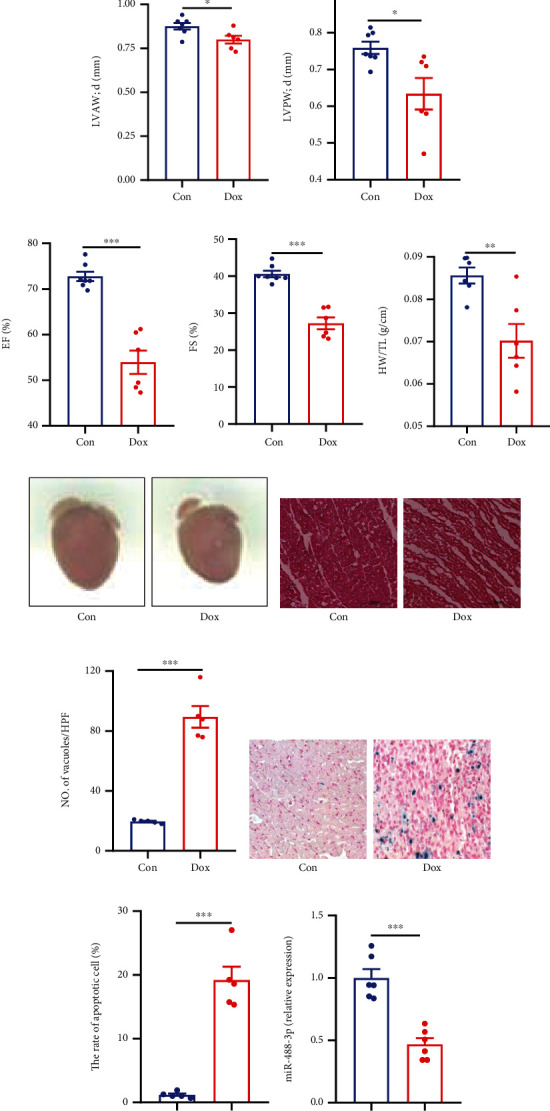
Doxorubicin induces myocardial injury with decreased miR-488-3p expression in vivo. (a) Schematic representation of the doxorubicin (Dox) treatment protocol in male C57BL/6 mice. (b–e) Echocardiographic (Echo) measurement of the left ventricular anterior wall (LVAW), left ventricular posterior wall (LVPW), ejection fraction (EF%), and fractional shortening (FS%) after doxorubicin treatment for 6 days (*n* = 6). (f) Heart weight- (HW-) to-tibia length (TL) ratios of mice in the control (Con) or Dox group (*n* = 6). (g) Representative image of the hearts of Con group and Dox group mice. (h) H&E staining of sections of mouse hearts of the Con group and Dox group. (i) The number of vacuoles was measured in the cytoplasm (*n* = 5). (j, k) The number of apoptotic cells was determined by terminal deoxynucleotidyl transferase-mediated dUTP nick-end labeling (TUNEL) staining in the mouse hearts of the Con group and Dox group. Blue staining shows TUNEL-positive cells (*n* = 5). (l) Relative expression of miR-488-3p in mouse left ventricles of the Con group and Dox group was determined by real-time qPCR (*n* = 6). ^∗^*P* < 0.05, ^∗∗^*P* < 0.01, and ^∗∗∗^*P* < 0.001.

Male C57BL/6 mice (10 weeks old) were intraperitoneally injected with doxorubicin (7.5 mg/kg) or saline every other day for 6 days (see Figure 2(a)). Echocardiography was utilized to detect the mouse cardiac systolic and diastolic functions. Compared with the control group, the structure of the left ventricle was impaired significantly among doxorubicin-treated groups. The wall thickness of the left ventricle was obviously thinner in the doxorubicin-treated group, as revealed by echocardiography analysis of LVAW and LVPW (see Figures 2(b) and 2(c)). Furthermore, consistent with heart structure change, EF% and FS% decreased significantly compared with the control group, indicating decreased cardiac systolic function after doxorubicin treatment (see Figures 2(d) and 2(e)). So, doxorubicin could induce abnormal heart structure and loss of heart function, which led to severe cardiotoxicity in vivo.

Compared with the control group, the hearts become smaller in doxorubicin-treated mice (see Figure 2(g)), as evidenced by quantification of the rate of heart weight to tibia length (see Figure 2(f)). The structure of the cardiac tissue was seriously disturbed in the doxorubicin-treated group as revealed by H&E staining (see Figure 2(h)). Besides, the morphology analysis of heart tissue also revealed obvious cytoplasmic vacuolization localized in the cardiac tissue treated with doxorubicin (see Figures 2(h) and 2(i)). As shown in Figure 2(j), the apoptotic cell rate increased significantly in the heart of doxorubicin-treated mice than the control mice revealed by TdT-mediated dUTP nick-end labeling (TUNEL) staining (see Figures 2(j) and 2(k)). Interestingly, compared with the control group, miR-488-3p levels decreased significantly in doxorubicin-treated mouse hearts compared with the control mouse hearts (see Figure 2(l)), suggesting that miR-488-3p might be involved in doxorubicin-induced myocardial toxicity in vivo.

### 3.3. miR-488-3p Expression Is Negatively Correlated with the Seriousness of Doxorubicin-Induced Cardiotoxicity

**Figure 3:**
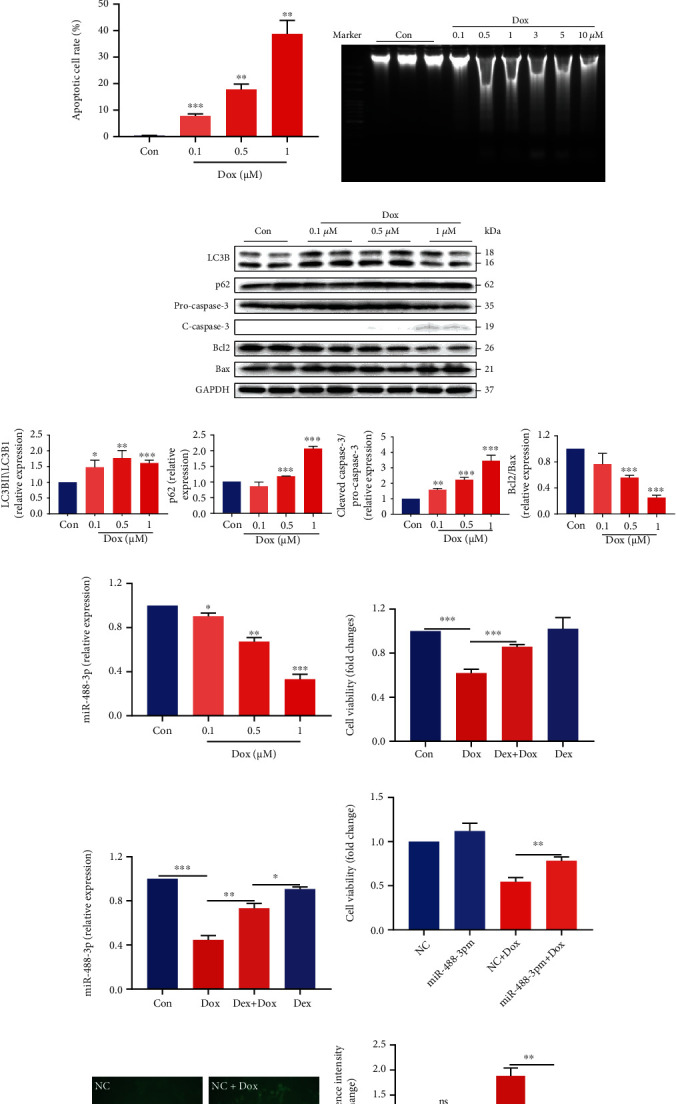
miR-488-3p expression is negatively correlated with the seriousness of doxorubicin-induced cardiotoxicity. (a) The cell viability of left ventricle cardiomyocytes treated with different concentrations of Dox for 24 hours, as demonstrated by the MTT assay (*n* = 4). (b, c) Cardiomyocytes were treated with Dox for 24 hours, and MDC staining images showing autophagosomes (b) and autophagy in cardiomyocytes were quantified (c) (*n* = 6). (d, e) Representative photos and the average data of cardiomyocyte apoptosis were analyzed by TUNEL staining in different concentrations of Dox treatment for 24 hours (*n* = 3). (f) DNA laddering of myocardial cells treated with Dox (0, 0.1 0.5, 1, 3, 5, and 10 *μ*M) for 24 hours. (g, h) Autophagy- and apoptosis-associated protein level in cardiomyocytes treated with different concentrations of Dox (0, 0.1, 0.5, and 1 *μ*M) for 24 h (*n* = 3). (i) Relative expression of miR-488-3p in cardiomyocytes with different concentrations of Dox was analyzed by qPCR (*n* = 3). (j) The viability of myocardial cells treated with Dox or dexrazoxane (Dex) alone or in combination (*n* = 4). (k) Levels of miR-488-3p were measured in myocardial cells treated with Dox or Dex alone or in combination (*n* = 4). (l) The viability of cardiomyocytes in the Con and 0.5 *μ*M Dox group after transfection with NC or miR-488-3p mimics (miR-488-3p m) (*n* = 4). (m, n) Fluorescence images showing NC- or miR-488-3p mimic-transduced cardiomyocytes that were treated with 0.5 *μ*M Dox or left untreated (m) and quantification of the MDC-stained autophagosomes (n) (*n* = 6). (o) Cardiomyocytes were transduced with NCi or miR-488-3p inhibitor (miR-488-3p i) and were treated with 0.5 *μ*M Dox or without Dox for 24 h. The formation of autophagosomes was examined using MDC staining after treatment with 0.5 *μ*M Dox (*n* = 5). ^∗^*P* < 0.05, ^∗∗^*P* < 0.01, and ^∗∗∗^*P* < 0.001.

To further investigate the mechanism of doxorubicin-induced cardiotoxicity, primary neonatal mouse ventricular myocardial cells were treated with different concentrations of doxorubicin. MTT assays were performed in different concentrations of doxorubicin-treated myocardial cells for 24 hours. As shown in Figure 3(a), consistent with the results in vivo, doxorubicin induced a concentration-dependent reduction in cell viability compared with controls. Neonatal mouse primary cardiomyocytes were treated with various concentrations of doxorubicin for 24 hours and analyzed by MDC staining. We identified that doxorubicin-induced autophagosomes significantly and concentration-dependently increased in cardiomyocytes (see Figure 3(b)), and the quantitative results are shown in Figure 3(c). TUNEL staining revealed that the number of apoptotic cardiomyocytes increased in a concentration-dependent manner in the doxorubicin-treated group compared with the control group (see Figures 3(d) and 3(e)). DNA laddering also demonstrated that different concentrations of doxorubicin induced different degrees of apoptosis in cardiomyocytes (see Figure 3(f)). Thus, doxorubicin could induce concentration-dependent autophagy and apoptosis in cardiomyocytes.

Then, we analyzed LC3B-II/LC3B-I and p62 level by Western blotting analysis. LC3B, a core contributor and marker to autophagy, mediates the elongation of the phagophore membrane. Consistent with prior reports, the levels of LC3B-II/LC3B-I and p62 increased significantly in a doxorubicin concentration-dependent manner in cardiomyocytes. The levels of apoptosis-related proteins such as cleaved caspase-3 and Bax also increased in a doxorubicin concentration-dependent manner (see Figures 3(g) and 3(h)). So, doxorubicin can induce both autophagic flux blockage and apoptosis in cardiomyocytes.

Interestingly, the expression of miR-488-3p decreased in a doxorubicin concentration-dependent manner in cardiomyocytes (see Figure 3(i)). Next, we use dexrazoxane, the only FDA-approved drug for preventing the cardiotoxicity of doxorubicin and improving cardiac function without affecting the antitumor activity of chemotherapy drugs, to improve heart function in doxorubicin-induced myocardial injury. Our results suggested that dexrazoxane attenuated doxorubicin-induced decline in cardiomyocyte viability (see Figure 3(j)). Next, we explored whether miR-488-3p is involved in the cardioprotective effect of dexrazoxane in doxorubicin-induced cardiotoxicity. As expected, compared with the doxorubicin-treated group, dexrazoxane could significantly increase miR-488-3p expression in doxorubicin-treated cardiomyocytes (see Figure 3(k)). Importantly, the MTT assay demonstrated that the cell viability notably increased upon overexpressing miR-488-3p (see Figure 3(l)).

On the other hand, we found no significant differences of autophagic vacuoles of cardiomyocytes when upregulating miR-488-3p, and doxorubicin-induced autophagy was blunted in miR-488-3p-overexpressing cardiomyocytes (see Figures 3(m) and 3(n)). In addition, to further confirm that miR-488-3p is involved in autophagy regulation in doxorubicin-treated cardiomyocytes, we next inhibited miR-488-3p by using the miR-488-3p inhibitor in cardiomyocytes. We identified that the miR-488-3p inhibitor increased the autophagy activity and further exacerbated doxorubicin-induced autophagy in cardiomyocytes (see Figure 3(o)). Altogether, our findings demonstrate that miR-488-3p indeed is one of the cardiomyocyte-protective mechanisms of dexrazoxane and attenuates doxorubicin-induced myocardial injury.

### 3.4. CyclinG1 Is an Important Downstream Target Gene of miR-488-3p in Cardiomyocytes

**Figure 4:**
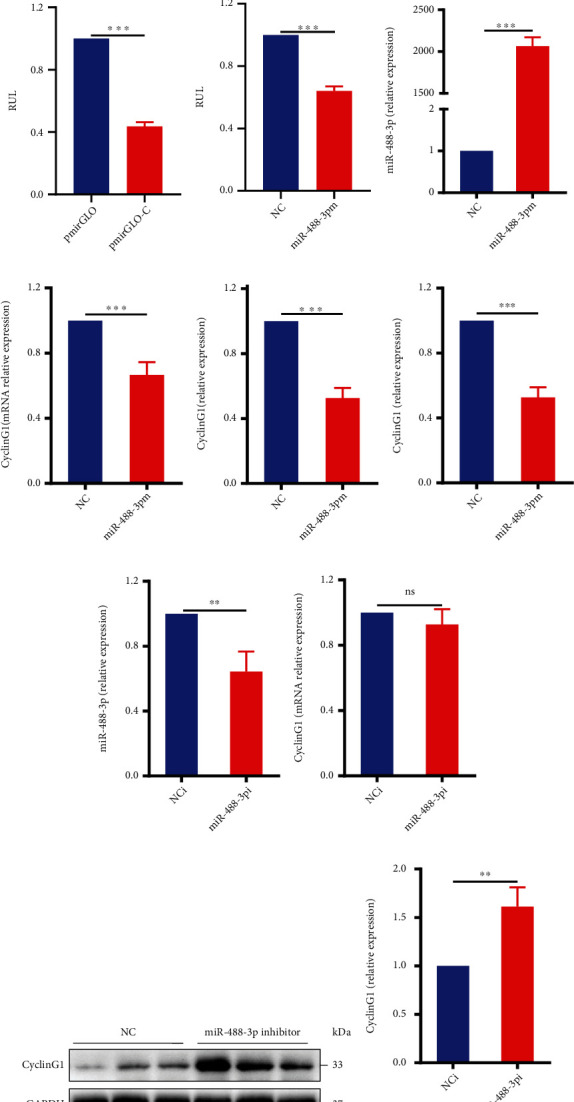
CyclinG1 is a downstream target gene of miR-488-3p. (a) The schematic of the miRNA-488-3p binding site to the target gene predicted and analyzed by using TargetScan. (b, c) Luciferase activity of pmirGLO-CyclinG1-3′-UTR (pmirGLO-C) in HEK 293A cells. The HEK 293A cells were cotransfected with pmirGLO, pmirGLO-CyclinG1-3′-UTR, and miR-488-3p mimic plasmid (*n* = 3). (d) Relative expression of miR-488-3p in cardiomyocytes after transfection with miR-488-3p mimics (*n* = 3). (e) The levels of CyclinG1 mRNA in cardiomyocytes after transfection with miR-488-3p mimics (*n* = 5). (f, g) Relative protein expression of CyclinG1 in cardiomyocytes after transfection with miR-488-3p mimics (*n* = 3). (h) The levels of miR-488-3p in cardiomyocytes after transfection with the miR-488-3p inhibitor (miR-488-3p i) (*n* = 3). (i) Relative mRNA expression of CyclinG1 in cardiomyocytes after transfection with the miR-488-3p inhibitor (*n* = 3). (j, k) The levels of CyclinG1 protein in cardiomyocytes after transfection with the miR-488-3p inhibitor (*n* = 3). ^∗∗^*P* < 0.01, ^∗∗∗^*P* < 0.001. ns indicates that no statistically significant difference was observed (*P* > 0.05).

Generally, miRNAs can negatively regulate its target gene expression by binding to miRNA recognition elements within the 3′-untranslated region (3′-UTR), which subsequently reduces mRNA stability, promotes mRNA degradation, or inhibits gene translation with posttranscription regulation [24]. In order to further explore the potential mechanism of myocardial protective effect of miRNA-488-3p, we predicted and analyzed the possible downstream target genes of miRNA-488-3p through online analysis software such as miRWalk, DianaBase, and TargetScan. Then, we verified the mRNA and protein functions of possible miR-488-3p's target genes and screened a downstream target gene: CyclinG1, which had dramatic changes and was directly related to cardiomyocyte function in vivo and in vitro. The results revealed specific binding sites between CyclinG1 and miR-488-3p (see Figure 4(a)).

To further identify the downstream target gene of miR-488-3p, luciferase activity of pmirGLO-CyclinG1-3′-UTR was performed in HEK 293A cells after cotransfection with the pmirGLO vector plasmid or pmirGLO-CyclinG1-3′-UTR plasmid. The sequencing results showed that the pmirGLO-CyclinG1-3′-UTR was correctly recombined into the plasmid pmirGLO. The recombinant plasmids pmirGLO-CyclinG1-3′-UTR, empty vector pmirGLO, miR-488-3p mimics, and its control miRNA (NC) were cotransfected into HEK 293A cells, and the fluorescence intensity of dual luciferase was analyzed. Luciferase activity was detected in the HEK293A cells after cotransfection with pmirGLO-CyclinG1-3′-UTR, vehicle control pmirGLO, and miR-488-3p mimics. The results showed that overexpression of miR-488-3p specifically reduced luciferase activity of pmirGLO-CyclinG1-3′-UTR in the HEK 293A cells compared with the control group (see Figures 4(b) and 4(c)). These results indicated that miR-488-3p could specifically bind to the 3′-UTR of CyclinG1.

We overexpressed miR-488-3p using chemically synthesized RNA mimics and knocked down its expression using a miR-488-3p inhibitor (an antisense sequence of nucleotide) in cardiomyocytes to investigate its function. After miR-488-3p overexpression (see Figure 4(d)), the mRNA and protein levels of CyclinG1 decreased significantly in cardiomyocytes as analyzed by real-time PCR and Western blot (see Figures 4(e)–4(g)). Moreover, miR-488-3p knockdown by the miR-488-3p inhibitor increased CyclinG1 expression significantly (see Figures 4(i)–4(k)). These results further proved that CyclinG1 is one of the miR-488-3p's downstream targets in cardiomyocytes.

### 3.5. CyclinG1 Knockdown Attenuates Doxorubicin-Induced Cardiomyocyte Autophagy and Apoptosis

**Figure 5:**
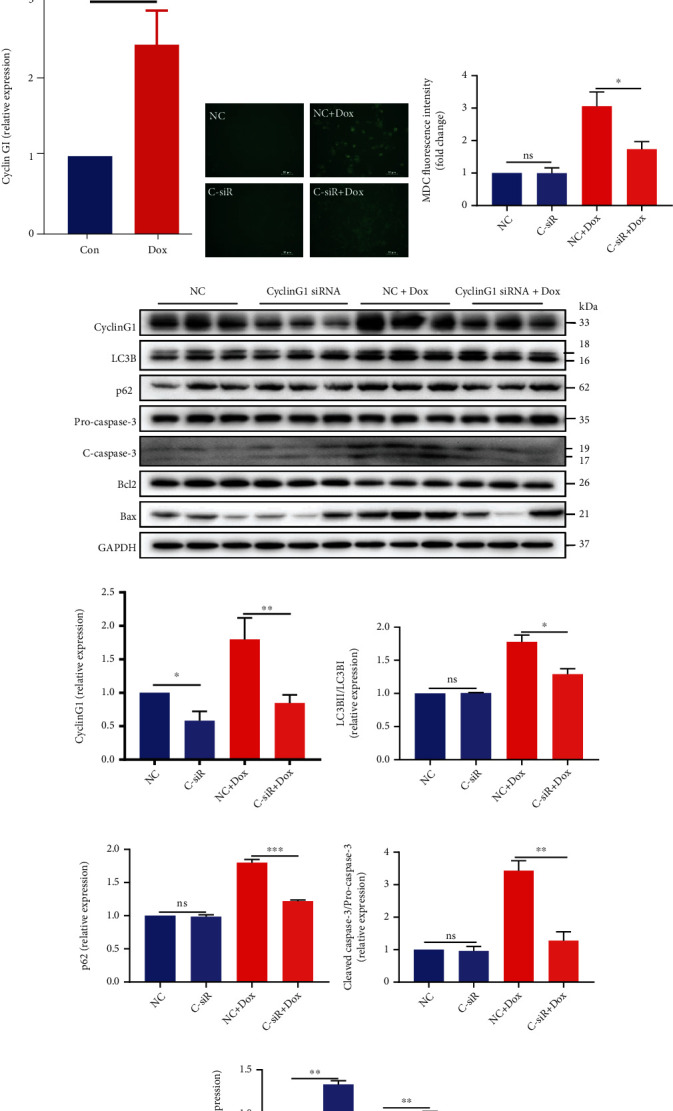
CylcinG1 knockdown attenuates doxorubicin-induced cardiotoxicity in vitro. (a) Relative mRNA expression of CyclinG1 in cardiomyocytes treated with 1 *μ*M Dox (*n* = 3). (b, c) Expression of CyclinG1 in control or 0.5 *μ*M Dox-treated cardiomyocytes (*n* = 3). (d) MDC staining of the cardiomyocytes treated for 24 h with and without 0.5 *μ*M Dox and after knockdown of CyclinG1 with siRNA or NC. (e) Graph showing the MDC fluorescence intensity quantified using the ImageJ program (*n* = 5). (f–k) The levels of CyclinG1 and autophagy- and apoptosis-associated protein expression were analyzed by Western blotting (*n* = 3). ^∗^*P* < 0.05, ^∗∗^*P* < 0.01. ns indicates that no statistically significant difference was observed (*P* > 0.05).

Next, we investigated CyclinG1's expression and function in doxorubicin-induced cardiomyocyte injury. In doxorubicin-treated cardiomyocytes, CyclinG1 increased significantly (see Figures 5(a)–5(c)). To further explore the potential function of CyclinG1 in doxorubicin-induced cardiotoxicity, we used CyclinG1 siRNA to knock down CyclinG1. As shown in Figure 5(d), MDC staining showed that CyclinG1 knockdown did not alter autophagic processes in cardiomyocytes in basal condition but significantly decreased autophagic response after doxorubicin treatment (see Figures 5(d) and 5(e)). Similarly, CyclinG1 knockdown did not change the LC3BII/I and p62 levels in basal condition but decreased the levels of LC3BII/I and p62 in doxorubicin-treated cardiomyocytes (see Figures 5(f)–5(i)), which suggested that CyclinG1 knockdown could inhibit doxorubicin-induced autophagic flux blockage in cardiomyocytes. These observations demonstrated that knockdown of CyclinG1 decreased doxorubicin-induced autophagosome accumulation in cardiomyocytes.

Furthermore, Western blotting revealed that cleaved caspase-3 decreased obviously in the CyclinG1 knockdown group compared with the control group after doxorubicin treatment (see Figures 5(f) and 5(j)). These results proved that the knockdown of CyclinG1 was sufficient to reduce the cardiomyocyte apoptosis induced by doxorubicin. Moreover, CyclinG1 knockdown also led to an increase in the Bcl2/Bax ratio in basal and doxorubicin-treated cardiomyocytes compared with wild-type CyclinG1 cardiomyocytes (see Figures 5(f) and 5(k)), which further supported that CyclinG1 knockdown could decrease doxorubicin-induced cardiomyocyte apoptosis and prolong cell survival.

## 4. Discussion

In the present study, we firstly provide new clues to prove the protective effect of miR-488-3p on doxorubicin-induced myocardial injury. Specifically, we have demonstrated that doxorubicin induces cardiotoxicity, which leads to cardiac structure abnormality and cardiac function loss. Remarkably, in myocardial cells treated with doxorubicin, miR-488-3p expression is downregulated significantly, which leads to autophagic flux blockage and cardiomyocyte apoptosis. Overexpression of miR-488-3p restores normal myocardial autophagy, reduces apoptosis, and attenuates doxorubicin-induced myocardial injury by inhibiting CyclinG1 expression.

### 4.1. Doxorubicin Causes Myocardial Injury and Heart Function Loss

At present, with the continuous progress of medical diagnosis and treatment methods and new drug research, cancer mortality is significantly reduced, while many complications averted or deracinated during cancer treatment remain unresolved, especially cardiovascular complications [25]. Therefore, looking for the effective ways to prevent or treat doxorubicin-induced cardiotoxicity becomes a hot topic in recent years, which also contributes to the development of oncocardiology [26]. The observation was reported for the first time in 1973 that doxorubicin may contribute to heart failure [27]. Indeed, heart failure was found in 30% of breast cancer patients in combination with trastuzumab and doxorubicin [28]. Our study reported that doxorubicin-induced cardiotoxicity is associated with the downregulation of miR-488-3p, which leads to heart structure abnormality, cardiac atrophy, and heart function loss. At the same time, we also demonstrated that the expression of miR-488-3p decreased in the hearts of doxorubicin-treated mice, indicating that miR-488-3p might mediate doxorubicin-induced myocardial injury.

### 4.2. Doxorubicin Leads to Concentration-Dependent Autophagic Flux Blockage and Cardiomyocyte Apoptosis

Doxorubicin is an efficacious chemotherapeutic drug commonly used for the treatment of hematologic malignancies, soft tissue sarcoma, and solid tumors [29]. The drug was first isolated from *S. peucetius* var. caesius by Arcamone et al. in 1969 in Italy [30]. But the clinical application of the drug is limited because of its acute and chronic cardiotoxic side effects [31]. The specific mechanism of doxorubicin-induced myocardial damage is not clear. Autophagy, as an early barrier or adaptive response of cells to stress conditions, is placed in the upstream of apoptosis and plays a critical role in maintaining cellular homeostasis. Studies have demonstrated that allopurinol can alleviate diabetes-induced myocardial injury and improve cardiac function through inhibiting the overactivated autophagy in rats [32]. On the other hand, it was reported that as an intracellular self-degraded pathway, autophagy could markedly inhibit cardiomyocyte senescence, attenuate ischemia/reperfusion-induced arrhythmic responses, and subsequently restore heart function by removing damaged organelles and proteins and recycling cytoplasmic constituents [33]. To further identify mechanisms by which doxorubicin elicits toxicity in cardiomyocytes, primary cardiomyocytes of neonatal mice were cultured in vitro and treated with doxorubicin. We found that doxorubicin could induce dose-dependent autophagic flux blockage and cardiomyocyte apoptosis in vivo and in vitro.

### 4.3. miR-488-3p Protects Cardiomyocytes against Doxorubicin-Induced Damage

Emerging data suggest a key regulatory effect of miR-488-3p in multiple tumors and other diseases. For example, the downregulation of miR-488-3p promoted epithelial-mesenchymal transition and cell migration of papillary thyroid cancer, suggesting the role of miR-488-3p as a tumor suppressor [17]. Moreover, miR-488-3p has been revealed to exhibit cancer-inhibiting effects by suppressing the expression of NOTCH2, such as significantly inhibiting the proliferation, invasion, and migration and cell cycle progression and inducing apoptosis in retinoblastoma [19]. In recent years, lncRNA myocardial infarction-associated transcript (MIAT) has been reported as an endogenous sponge to absorb miR-488-3p, which subsequently inhibits the effects of miR-488-3p and regulates gene expression [18]. Emerging data suggest that MIAT, a novel lncRNA related to myocardial infarction, is important for cardiomyocyte survival and apoptosis, as well as regulation of cardiac function under pathophysiological conditions. For example, MIAT was abundantly expressed in myocardial infarction. Silencing MIAT significantly decreased apoptosis and increased ATP production in cardiomyocytes, thus alleviating the impairment of cardiac function after myocardial infarction [34]. Previous studies have shown that miR-488 can inhibit the expression of proinflammatory cytokines in gouty arthritis by targeting the 3′-UTR of IL-1*β*. The upregulation of miR-488 inhibits monosodium urate-induced expression of IL-1*β* proteins in THP-1 cells and plays a posttranscriptional regulatory role in gouty arthritis [35]. Although miR-488-3p has been studied in different cancer types and other diseases, there are few published studies investigating the function and regulatory mechanisms of miR-488-3p in doxorubicin-induced cardiotoxicity. In our study, the expression of miR-488-3p was significantly downregulated in a concentration-dependent manner in doxorubicin-treated cardiomyocytes. Subsequently, to further explore the role of miR-488-3p in doxorubicin-induced cardiotoxicity, miR-488-3p mimics or inhibitor was transfected into primary cardiomyocytes to overexpress or knockdown miR-488-3p. We found that overexpression of miR-488-3p inhibited autophagy and cell viability decrease induced by doxorubicin, which suggested that miR-488-3p might protect cardiomyocytes from doxorubicin-induced cardiotoxicity. Next, to verify its cardioprotective function, we further investigated the effect of downregulation of miR-488-3p in cardiomyocytes treated with doxorubicin. We found that the miR-488-3p inhibitor induced the formation of autophagosomes and further aggravated doxorubicin-induced autophagy in primary cardiomyocytes. Taken together, these results suggest that miR-488-3p has a protective effect on cardiomyocytes and suggest that miR-488-3p can suppress doxorubicin-induced autophagic flux blockage in primary cardiomyocytes.

### 4.4. miR-488-3p Attenuates Doxorubicin-Induced Myocardial Damage via Suppression of CyclinG1

CyclinG1, a member of the CyclinG family, is homologous to the c-src [36]. It has been demonstrated that CyclinG1 is not a typical cyclin and has no counterpart in cyclin-dependent kinases. Therefore, CyclinG1 is considered an orphan cyclin and has not been reported to play an important role in cell cycle regulation, but recent studies have shown that it is an important downstream target gene for p53 and has been suggested to be involved in the regulation and stability of p53 [37]. It has been reported that CyclinG1 is an important negative modulator of the p53-MDM2 pathway by reducing p53 stability and increasing p53 degradation [38]. CyclinG1 is also the only cyclin that can be activated by p53 at the transcriptional level and has two functional binding sites that can bind to transcription factors [39].

Genetic function analysis showed that CyclinG1 was closely related to apoptosis-related pathways. Yet, the regulatory role of CyclinG1 in cell growth is still controversial and inconclusive. Some studies have shown that the overexpression of CyclinG1 can enhance the proliferation of cancer cells, and the inhibition of CyclinG1 inhibits cell growth, indicating that CyclinG1 promotes cell growth [40]. Similarly, CyclinG1 expression increases in some tumors such as myeloma, breast cancer, and leiomyoma. In contrast, some research has been reported that CyclinG1 stability [41] and expression increase after DNA damage, indicating that CyclinG1 has an inhibitory effect on cell growth [42]. Moreover, studies have demonstrated that CyclinG1's effect on growth depends on its level of expression. Low level of CyclinG1 does not inhibit cell growth (or even promote cell growth), and overexpression of CyclinG1 inhibits cell growth and causes apoptosis [43]. CyclinG1 also plays a different role in proliferating cells and terminally differentiated cells [37]. However, the function of CyclinG1 in cardiomyocytes is still not clear. Here, we demonstrated that doxorubicin could lead to upregulation of CyclinG1 and promote autophagy and cell apoptosis. Interestingly, we also recognized that CyclinG1 is one of the downstream target genes of miR-488-3p, which can inhibit autophagy and apoptosis and alleviate doxorubicin-induced cardiotoxicity.

Indeed, previous studies have suggested that various pathologic conditions, such as toxins, medications, hypertension, diabetes, and obesity, might trigger cardiomyocyte autophagy and apoptosis, cause disordered cardiac structure, and then lead to cardiac dysfunction [44–47]. Therefore, our findings will provide a new perspective and novel therapeutic targets for effectively alleviating myocardial injury.

## 5. Conclusions

Taken together, our study reveals the heart-protective effect of miR-488-3p in doxorubicin-induced cardiotoxicity. We demonstrate that miR-488-3p improves cardiac function and reduces doxorubicin-induced cardiomyocyte autophagic flux blockage and apoptosis by inhibiting the expression of CyclinG1, which provides a theoretic target for preventing heart injury induced by doxorubicin. The therapeutic strategies that directly target miR-488-3p and CyclinG1 may represent an innovative way to limit doxorubicin-induced cardiotoxicity in the future.

## Data Availability

All data needed to evaluate the conclusions in the paper are present in the paper. Additional data related to this paper may be requested from the authors.

## References

[1] Kocarnik J. M., Compton K., Dean F. E. (2021). Cancer incidence, mortality, years of life lost, years lived with disability, and disability-adjusted life years for 29 cancer groups from 2010 to 2019: a systematic analysis for the Global Burden of Disease Study 2019. *JAMA Oncology*.

[2] Miller K. D., Nogueira L., Mariotto A. B. (2019). Cancer treatment and survivorship statistics, 2019. *CA: a Cancer Journal for Clinicians*.

[3] Zhang Y. Y., Yi M., Huang Y. P. (2017). Oxymatrine ameliorates doxorubicin-induced cardiotoxicity in rats. *Cellular Physiology and Biochemistry*.

[4] Kirkham A. A., Eves N. D., Shave R. E. (2018). The effect of an aerobic exercise bout 24 h prior to each doxorubicin treatment for breast cancer on markers of cardiotoxicity and treatment symptoms: a RCT. *Breast Cancer Research and Treatment*.

[5] Georgakopoulos P., Kyriakidis M., Perpinia A. (2019). The role of metoprolol and enalapril in the prevention of doxorubicin-induced cardiotoxicity in lymphoma patients. *Anticancer Research*.

[6] Zhang W. B., Lai X., Guo X. F. (2021). Activation of Nrf2 by miR-152 inhibits doxorubicin-induced cardiotoxicity via attenuation of oxidative stress, inflammation, and apoptosis. *Oxidative Medicine and Cellular Longevity*.

[7] Benjanuwattra J., Siri-Angkul N., Chattipakorn S. C., Chattipakorn N. (2020). Doxorubicin and its proarrhythmic effects: a comprehensive review of the evidence from experimental and clinical studies. *Pharmacological Research*.

[8] Nam J. K., Kim A. R., Choi S. H. (2021). An antibody against L1 cell adhesion molecule inhibits cardiotoxicity by regulating persistent DNA damage. *Nature Communications*.

[9] Galan-Arriola C., Villena-Gutiérrez R., Higuero-Verdejo M. I. (2021). Remote ischaemic preconditioning ameliorates anthracycline-induced cardiotoxicity and preserves mitochondrial integrity. *Cardiovascular Research*.

[10] Li D. L., Wang Z. V., Ding G. (2016). Doxorubicin blocks cardiomyocyte autophagic flux by inhibiting lysosome acidification. *Circulation*.

[11] Zilinyi R., Czompa A., Czegledi A. (2018). The cardioprotective effect of metformin in doxorubicin-induced cardiotoxicity: the role of autophagy. *Molecules*.

[12] Yu X., Ruan Y., Shen T. (2020). Dexrazoxane protects cardiomyocyte from doxorubicin-induced apoptosis by modulating miR-17-5p. *BioMed Research International*.

[13] Yu X., Ruan Y., Huang X. (2020). Dexrazoxane ameliorates doxorubicin-induced cardiotoxicity by inhibiting both apoptosis and necroptosis in cardiomyocytes. *Biochemical and Biophysical Research Communications*.

[14] Kim S. Y., Kim S. J., Kim B. J. (2006). Doxorubicin-induced reactive oxygen species generation and intracellular Ca2+ increase are reciprocally modulated in rat cardiomyocytes. *Experimental & Molecular Medicine*.

[15] Baek D., Villén J., Shin C., Camargo F. D., Gygi S. P., Bartel D. P. (2008). The impact of microRNAs on protein output. *Nature*.

[16] Wang Q., Yu X., Dou L. (2019). miR-154-5p functions as an important regulator of angiotensin II-mediated heart remodeling. *Oxidative Medicine and Cellular Longevity*.

[17] Zhang W., Liu T., Li T., Zhao X. (2021). Hsa *circRNA*102002 facilitates metastasis of papillary thyroid cancer through regulating miR-488-3p/HAS2 axis. *Cancer Gene Therapy*.

[18] Liu Y., Peng H., Shen Y., Da R., Tian A., Guo X. (2020). Downregulation of long noncoding RNA myocardial infarction associated transcript suppresses cell proliferation, migration, invasion, and glycolysis by regulation of miR-488-3p/IGF1R pathway in colorectal cancer. *Cancer Biotherapy & Radiopharmaceuticals*.

[19] Wu X. Z., Cui H. P., Lv H. J., Feng L. (2019). Knockdown of lncRNA PVT1 inhibits retinoblastoma progression by sponging miR-488-3p. *Biomedicine & Pharmacotherapy*.

[20] Wen Z., Shen Q., Zhang H. (2019). Circular RNA CCDC66 targets DCX to regulate cell proliferation and migration by sponging miR-488-3p in Hirschsprung's disease. *Journal of Cellular Physiology*.

[21] Sirey T. M., Roberts K., Haerty W. (2019). The long non-coding RNA Cerox1 is a post transcriptional regulator of mitochondrial complex I catalytic activity. *eLife*.

[22] Shen T., Aneas I., Sakabe N. (2011). Tbx20 regulates a genetic program essential to adult mouse cardiomyocyte function. *The Journal of Clinical Investigation*.

[23] Shen T., Zheng M., Cao C. (2007). Mitofusin-2 is a major determinant of oxidative stress-mediated heart muscle cell apoptosis. *The Journal of Biological Chemistry*.

[24] Bushati N., Cohen S. M. (2007). MicroRNA functions. *Annual Review of Cell and Developmental Biology*.

[25] Okwuosa T. M., Prabhu N., Patel H. (2018). The cardiologist and the cancer patient: challenges to cardio-oncology (or onco-cardiology) and call to action. *Journal of the American College of Cardiology*.

[26] Zamorano J. L., Lancellotti P., Rodriguez Muñoz D. (2017). 2016 ESC Position Paper on cancer treatments and cardiovascular toxicity developed under the auspices of the ESC Committee for Practice Guidelines: the Task Force for cancer treatments and cardiovascular toxicity of the European Society of Cardiology (ESC). *European Journal of Heart Failure*.

[27] Lefrak E. A., Piťha J., Rosenheim S., Gottlieb J. A. (1973). A clinicopathologic analysis of adriamycin cardiotoxicity. *Cancer*.

[28] Shum K., Solivan A., Parto P., Polin N., Jahangir E. (2016). Cardiovascular risk and level of statin use among women with breast cancer in a cardio-oncology clinic. *The Ochsner Journal*.

[29] Niu W., Xiao Q., Wang X. (2021). A biomimetic drug delivery system by integrating grapefruit extracellular vesicles and doxorubicin-loaded heparin-based nanoparticles for glioma therapy. *Nano Letters*.

[30] Arcamone F., Cassinelli G., Fantini G. (2000). Adriamycin, 14-hydroxydaunomycin, a new antitumor antibiotic from *S. peucetius var. caesius*. *Reprinted from Biotechnology and Bioengineering*.

[31] Tanaka Y., Nagoshi T., Yoshii A. (2021). Xanthine oxidase inhibition attenuates doxorubicin-induced cardiotoxicity in mice. *Free Radical Biology & Medicine*.

[32] Luo J., Yan D., Li S. (2020). Allopurinol reduces oxidative stress and activates Nrf2/p62 to attenuate diabetic cardiomyopathy in rats. *Journal of Cellular and Molecular Medicine*.

[33] Lekli I., Haines D. D., Balla G., Tosaki A. (2017). Autophagy: an adaptive physiological countermeasure to cellular senescence and ischaemia/reperfusion-associated cardiac arrhythmias. *Journal of Cellular and Molecular Medicine*.

[34] Cao X., Ma Q., Wang B. (2021). Silencing long non-coding RNA MIAT ameliorates myocardial dysfunction induced by myocardial infarction via MIAT/miR-10a-5p/EGR2 axis. *Aging (Albany NY)*.

[35] Zhou W., Wang Y., Wu R., He Y., Su Q., Shi G. (2017). MicroRNA-488 and -920 regulate the production of proinflammatory cytokines in acute gouty arthritis. *Arthritis Research & Therapy*.

[36] Tamura K., Kanaoka Y., Jinno S. (1993). Cyclin G: a new mammalian cyclin with homology to fission yeast Cig1. *Oncogene*.

[37] Liu Z., Yue S., Chen X., Kubin T., Braun T. (2010). Regulation of cardiomyocyte polyploidy and multinucleation by cyclinG1. *Circulation Research*.

[38] Ohtsuka T., Ryu H., Minamishima Y. A., Ryo A., Lee S. W. (2003). Modulation of p53 and p73 levels by cyclin G: implication of a negative feedback regulation. *Oncogene*.

[39] Zauberman A., Lupo A., Oren M. (1995). Identification of p53 target genes through immune selection of genomic DNA: the cyclin G gene contains two distinct p53 binding sites. *Oncogene*.

[40] Skotzko M., Wu L., Anderson W. F., Gordon E. M., Hall F. L. (1995). Retroviral vector-mediated gene transfer of antisense cyclin G1 (CYCG1) inhibits proliferation of human osteogenic sarcoma cells. *Cancer Research*.

[41] Li H., Okamoto K., Peart M. J., Prives C. (2009). Lysine-independent turnover of cyclin G1 can be stabilized by B'*α* subunits of protein phosphatase 2A. *Molecular and Cellular Biology*.

[42] Shimizu A., Nishida J. I., Ueoka Y. (1998). CyclinG contributes to G2/M arrest of cells in response to DNA damage. *Biochemical and Biophysical Research Communications*.

[43] Zhao L., Samuels T., Winckler S. (2003). Cyclin G1 has growth inhibitory activity linked to the ARF-Mdm2-p53 and pRb tumor suppressor pathways. *Molecular Cancer Research*.

[44] Yan M., Sun S., Xu K. (2021). Cardiac aging: from basic research to therapeutics. *Oxidative Medicine and Cellular Longevity*.

[45] Gellen B., Thorin-Trescases N., Sosner P. (2016). ANGPTL2 is associated with an increased risk of cardiovascular events and death in diabetic patients. *Diabetologia*.

[46] Demaison L. (2020). Oxidative stress and obesity- and type 2 diabetes-induced heart failure. *Antioxidants (Basel)*.

[47] Li N., Zhou H. (2020). SGLT2 inhibitors: a novel player in the treatment and prevention of diabetic Cardiomyopathy. *Drug Design, Development and Therapy*.

